# Dispersal of *Bacillus subtilis* and its effect on strawberry phyllosphere microbiota under open field and protection conditions

**DOI:** 10.1038/srep22611

**Published:** 2016-03-03

**Authors:** Feng Wei, Xiaoping Hu, Xiangming Xu

**Affiliations:** 1 State Key Laboratory of Crop Stress Biology for Arid Areas, College of Plant Protection, Northwest A&F University, Taicheng Road 3, Yangling 712100, China; 2 NIAB East Malling Research, East Malling, Kent, ME19 6BJ, UK

## Abstract

Using biological control agents (BCAs) is an essential component of integrated pest and diseases management. Despite much research on biocontrol of plant diseases, success in field crops has been limited with most successes being achieved in greenhouse cultivation. This lack of success is often attributed to the complex ecological processes involved in biocontrol. We used next generation sequencing (NGS) technology to study environmental fate of *Bacillus subtilis*, a widely used BCA, focusing on its dispersal aspect in open field and under protection. The dispersal of *B. subtilis* was very limited, particularly under protection. The reduction in the BCA population size was relatively small within 8 days; indeed, no overall reduction in the relative abundance was observed under the protected condition. These results suggested that limited dispersal is probably the main reason for its variable (and often low) control efficacy under field conditions. Thus to increase biocontrol efficacy, it is necessary to frequently apply this BCA with the application interval depending on the growth rate of target host tissues. Phyllosphere microbiota differed significantly between plants grown in open field and under protection but were not greatly affected by the introduced BCA.

The use of biological control agents (BCAs) to manage plant diseases is important for crop production as the number of available effective synthetic fungicides reduces rapidly because of government regulations and development of pathogen resistance to fungicides^1^,^2^. A number of beneficial microorganisms have been studied for controlling plant diseases, such as *Bacillus* spp., *Agrobacterium radiobacter*, *Ampelomyces quisqualis, Aspergillus flavus, Coniothyrium minitans, Gliocladium Catenulatum, Pseudomonas* spp.*, Trichoderma harzianum* etc. However, because of the complex ecological processes involved, success in biocontrol of plant diseases in field crops has been limited^3^; most successes have been achieved in greenhouse cultivation^4^,^5^,^6^,^7^. Understanding ecological characteristics of BCAs will assist us in developing strategies to maximise their efficacies in field conditions^8^,^9^,^10^. Although a *Trichoderma atroviride* isolate can survive on strawberry leaves under greenhouse conditions for 45 days^11^, population size of BCAs usually decline rapidly to a lower level after several days^5^,^12^. Thus, foliar BCA applications have to be sprayed frequently to maintain and an active population.

Recent modelling studies show biocontrol outcomes critically depend on the rate of BCA colonizing healthy and diseased host tissues, as well as BCA mortality^2^,^13^,^14^,^15^. Unfortunately, there is little published direct information on these, especially on the rate of BCA colonising healthy tissue. Two aspects of the rate of BCA colonising healthy tissues need to be considered: the colonisation of immediate neighbouring tissues in the same host tissue unit and the colonisation of (i.e. dispersed to or spreading to) new tissue units. Only by the latter route, can a BCA fully realise the often-claimed advantage of a BCA being able to colonise new host tissues. It should be noted that the requirement for a BCA to spread to and colonise new tissues is not necessary for some biocontrol systems, such as post-harvest application of BCAs to control storage diseases. The dispersal aspect of biocontrol of foliar pathogens has been rarely studied probably because it has been accepted that BCAs have to be repeatedly applied in order to control foliar pathogens satisfactorily. However, the interval of application depends critically on plant growth rate and the type of tissues that need to be protected. For instance, more frequent applications are needed in order to protect flowers if new flowers are continually opening, especially for those crops with long flowering/fruiting period, e.g. day-neutral strawberries.

*Bacillus* spp. are globally dispersed bacteria producing numerous bioactive compounds with a broad spectrum of activities towards pathogens or inducing host systemic resistance^16^,^17^,^18^,^19^. A biocontrol product Serenade, formulated of a single *B. subtilis* isolate QST713, is probably the most widely used biocontrol product of plant diseases worldwide; it is reported to be effective in controlling many foliar and soilborne pathogens. However, observed efficacies are not always consistent. For example, Serenade treatment suppressed clubroot on canola under controlled conditions, but its performance varied greatly under field conditions^7^.

Strawberry is an important high-value crop. Grey mould on fruit, caused by *Botrytis cinerea*, is a major disease of strawberry^20^. In the UK, in order to mitigate the threat of soilborne disease and to extend the season, more than half of strawberry production is now produced in substrate under protection where powdery mildew is becoming a serious problem. Serenade is used to control both diseases but with extremely variable (often low) efficacies (Xu, X. M. unpublished work). We conducted studies to specifically assess the extent of dispersal of *B. subtilis* among strawberry leaves in both open field and under protection conditions. Relative population sizes of phyllosphere microbiota, including *B. subtilis*, were quantified based on a meta-barcoding approach using next generation sequencing (NGS) technology. Using the data, we also assessed the differences in the phyllosphere microbiota between open field and under protection, and determined which microbes were affected by the introduced *B. subtilis*. The composition of phyllosphere communities could be affected by numerous abiotic and biotic factors, including invading microorganisms^8^,^21^,^22^,^23^,^24^.

## Results

### Sequencing quality and picking OTUs

All samples except one generated a sufficient number of reads that passed the initial quality check. The only sample that failed was for the ITS data of the treatment combination – untreated control from protected plants ‘New’ sample. The number of merged 16S rDNA sequences that were assigned to operational taxonomic units (OTUs) ranged from 25,700 to 220,000 with a median of 94,600; the number of fungal ITS sequences that were assigned to OTUs ranged from 19,400 to 134,000 with a median of 63,200. There were 67 and 856 bacterial and fungal OTUs, respectively. Rarefaction analysis showed that the present sequencing depth appeared to be sufficient for all samples (supplementary Fig. S1).

Mapping the OTUs into known taxonomy units was much less certain for bacteria than for fungi. For 22 of 67 bacterial OTUs, the probability of correct classification into the phylum was less than 50%. The lower the taxonomy rank, the lower the confidence in the classification. On the other hand, classification of fungal OTUs into known taxonomy ranks was more certain, particularly for high ranks; even for the genus level, P-values were greater than 50% for nearly all OTUs. Consequently, about one third of bacterial OTUs were classified as ‘Unknown’ at the phylum level, compared to 2% for fungi [i.e. for these OTUs, P-values at the phylum level were <95%]. There were 6, 3, 1, 35 and 22 OTUs in the Actinobacteria, Bacteroidetes, Firmicutes, ‘Unknown’, and Proteobacteria categories, respectively. The only Firmicutes OTU was *Bacillus sp.* and was confirmed to be *B. subtilis* after further validation via BLAST search in the NCBI database – this OTU shared 99% identity with many *B. subtilis* strains (e.g. Accessions AM882680, KR296931.1 and KR336829.1). There were 709, 128, 5 and 12 fungal OTUs in the Ascomycota, Basidiomycota, Chytridiomycota, and ‘Unknown’ categories, respectively.

### Diversity

**Figure 1:**
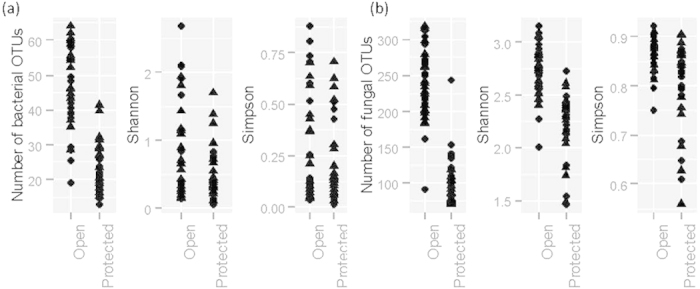
Average α diversity measures for strawberry phyllosphere microbiota. They were calculated from bacterial (**a**) and fungal (**b**) OTUs of 72 strawberry leaf samples based on 100 bootstrap samples (re-sample size = 25,000 for bacteria and 19,000 for fungi): number of observed OTUs, and Shannon and Simpson indices. Different symbols represent three leaf samples.

**Figure 2:**
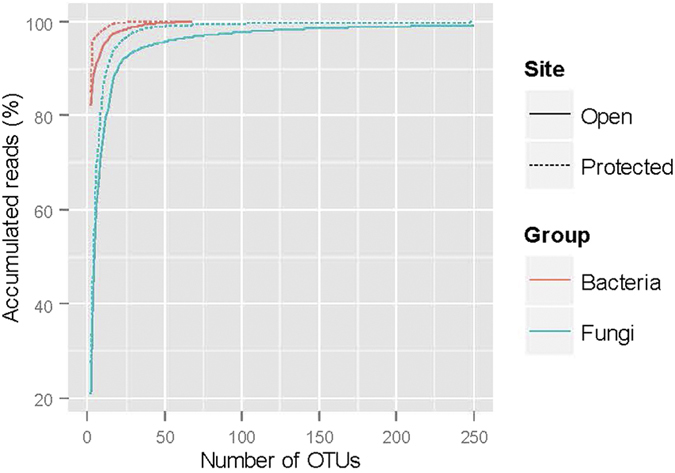
The accumulated number of reads. The data were expressed as the percentage of the total reads in relation to the number of bacterial and fungal OTUs in 36 strawberry leaves of cv. Vibrant grown in the open field or under protection. Half of the samples were treated with Serenade (a commercial formulated product of *Bacillus subtilis*) and the other half without.

Within-sample diversity measures varied greatly with samples (Figs 1 and 2). A single OTU in the ‘Unknown’ category accounted for more than 80% of the total bacterial reads (Fig. 2). Blast of the sequence of this OTU suggested that it could be the DNA from strawberry chloroplast but could also from an uncultured bacterium. On average, the diversity indices were higher for samples from the open field than from the tunnel (P < 0.001) and for new leaves than for old leaves (P < 0.05). For example, average number of bacterial OTUs was 47 and 23 for the open and protected plantings, respectively, although all bacterial OTUs were present across all samples from the protected plants. There were 20 (or more as the ‘Unknown’ family may contain several taxonomic families) bacterial families detected in the samples; Sphingomonadaceae accounted for 91.9% and 97.1% of the total reads for the open and protected plant samples, respectively. Next is the ‘Unknown’ category, accounting for the respective 5.7% and 2.4% of the total reads. Average number of bacterial OTUs was 31, 32 and 42 for the ‘4H’, ‘8D’ and ‘New’ leaf samples, respectively. There were no significant differences in these indices between ‘BCA’ and ‘Control’ treatments.

For fungi, the α diversity indices were greater (P < 0.001) for samples from the open than from the protected plants (Fig. 1b). For instance, there were on average 238 and 103 fungal OTUs for the open and protected plant samples, respectively, although nearly all fungal OTUs that were detected in the open samples were also present in one or more samples from the protected plants. Fungal OTUs were from 90 families (or more as the ‘Unknown’ family may contain several taxonomic families); there were considerable differences in the proportion of reads from most common families. There were proportionally more reads of the ‘Unknown’ (70.4%) and Gnomoniaceae (9.8%) in the protected samples than in the open samples (65.0% and 2.3%); the opposite is true for Filobasidiaceae (24.0% for the open and 16.2% for the protected) and Pucciniastraceae (7.0% for the open and 1.1% for the protected). The other 86 families only accounted for 1.7% and 2.4% of reads for the open and protected samples, respectively. The effect of different types of leaf samples on the indices was not consistent as for bacteria.

**Figure 3:**
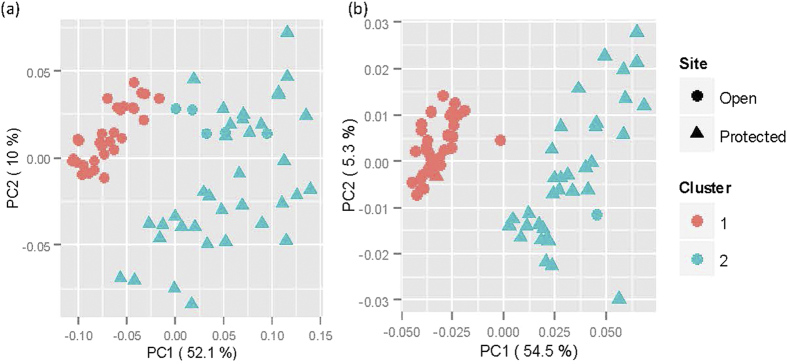
Principal component analysis of microbial communities on strawberry leaves. First two Principle Components derived from correlation in individual OTU abundance among individual samples for bacteria (**a**) and fungi (**b**) – the OTU abundance was calculated as log_2_(x_*i*_ + 1)/∑(log_2_(x_*i*_ + 1)) for a given sample where x is the original counts value for the *i*th OTU. The two clusters were derived from the weighted UniFrac distance using the Ward method. Strawberry plants of cv. Vibrant grown in the open or under protection were sprayed or not sprayed with Serenade (a commercial formulated product of *Bacillus subtilis*); leaves were sampled 4 hours (4H) and 8 days (8D) after spraying. In addition, leaves emerged after spraying were also sampled on day 8. Microbial populations on the phyllosphere were profiled with the Illumina MiSeq.

Clustering analysis of UniFrac distances and principal component analysis of the correlation for both bacteria and fungi showed that samples separated in two groups, largely corresponding to the open and protected conditions (Fig. 3).

### Differential abundance of microbes between two environments

For bacteria, only 38 OTUs were left after automatic filtering by DESeq2; for 22 of these OTUs, there were significant differences (BH adjusted P < 0.05) in bacterial abundance between the open and protected plant samples, with 1, 1, 13 and 8 OTUs from respective Actinobacteria, Firmicutes, ‘Unknown’, and Proteobacteria. For 14 of the 22 OTUs, the abundance is greater for samples from the open than from protected plants. Abundance of *Bacillus*, *Pseudomonas* and *Xanthomonas* was all less for the open than for the protected plant samples.

For fungi, there were 306 OTUs left after automatic filtering by DESeq2; for 169 of these OTUs, there were significant differences (BH adjusted P < 0.05) in the abundance between the open and protected plant samples, with 134, 34 and 1 OTUs from respective Ascomycota, Basidiomycota and ‘Unknown’ category. For 146 of the 169 OTUs, the abundance is greater for samples from the open than from protected plants.

### Analysis of *B. subtilis* abundance

**Figure 4:**
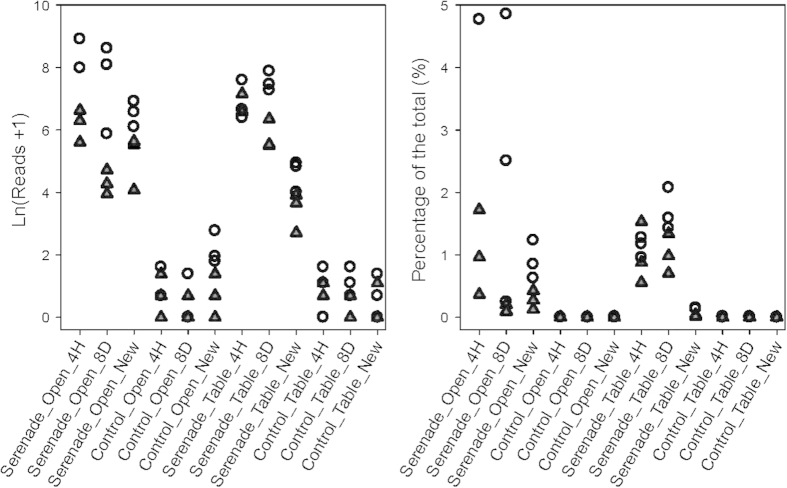
Number of sequence reads of *Bacillus subtilis* (ln transformed) for all individual samples. Strawberry plants of cv. Vibrant grown in the open or under protection were sprayed with Serenade (a commercial formulated product of *Bacillus subtilis*) or not (Control); leaves were sampled 4 hours (4H) and 8 days (8D) after spraying. In addition, leaves emerged after the spraying (New) were also sampled on day 8. The triangle and circle points represent two replicate experiments. Microbial populations on the phyllosphere were profiled with the Illumina MiSeq.

The abundance of *B. subtilis* increased greatly following the application, and declined 8 days after, and there was also an appreciable amount of *B. subtilis* on new leaves (Fig. 4, Table 1). Application of Serenade led to the increased (P < 0.001) abundance of *B. subtilis* when assessed four hours after application; there were only 3 reads in the untreated samples, compared to 2,014 reads in the BCA-treated samples. Even 8 days after the applications, there was still much greater (P < 0.001) abundance of *B. subtilis* on the treated ‘8D’ leaves (1,355 reads) than on the ‘control’ leaves (1 read). However, this difference was greater (P < 0.001) for the ‘protected’ than for the open plant samples. Similarly there was large loss (P < 0.05) in *B. subtilis* during the 8 days, depending on the environment where the sample came from (Fig. 4): *B. subtilis* abundance reduced by ca. 50% for the open plant samples whereas the abundance on protected plants did not change much.

The abundance of *B. subtilis* on the ‘New’ leaves (emerged after the BCA application) was greater (P < 0.001) for the treated (264 reads) than the control (3 reads) samples, irrespective of the plantations. However, the abundance difference between the ‘4H’ and ‘New’ samples was greater (P < 0.001) for the protected than for the open plant samples (Table 1). The number of *B. subtilis* reads in the ‘New’ samples was about 15% of that in the ‘4H’ samples for the open, compared with the 7% for the protected plants.

### Effects of *B. subtilis* on individual microbial OTUs

On the ‘8D’ leaf samples, only 2 of 48 bacterial OTUs (after filtering) differed (BH adjusted P < 0.05) in their abundance between the BCA treated and the control samples: the control samples had lower abundance of *B. subtilis* than the treated samples; the opposite was true for *Sphingomonas* OTU. Only 2 out of 13 OTUs (after filtering) differed (BH adjusted P < 0.05) in their abundance on the ‘New’ leaf samples between the BCA treatments: abundance of *B. subtilis* was greater in the treated samples than in the control samples; the opposite was true for an OTU from the ‘Unknown’ category.

On the ‘8D’ leaf samples, only 8 of 183 fungal OTUs (after filtering) differed (BH adjusted P < 0.05) in their abundance between the BCA treated and the control samples: six were from Ascomycota and the others from Basidiomycota. For six samples (including a powdery mildew OTU from *Blumeria*), the control samples had greater abundance than the treated samples. Only 1 of 285 OTUs (after filtering) differed (BH adjusted P < 0.05) in its abundance on the ‘New’ leaf samples between the BCA treatments: abundance of a *Cryptococcus* OTU was greater in the control samples than in the treated samples – this difference was also significant on the ‘8D’ leaf samples.

## Discussion

We used NGS to study the environmental fate of *B. subtilis*, a widely used biocontrol agent of plant pathogens, focusing on its dispersal aspect. The dispersal of *B. subtilis* was shown to be very limited, particularly under the protected condition. The reduction in its population size was relatively small within 8 days of application; indeed, no overall reduction in its abundance was observed for the protected condition. These results suggest that limited dispersal is probably the main reason for its variable (and often low) control efficacy under field conditions.

NGS as a semi-quantitative method only allows quantification of the relative abundance of different OTUs^25^, rather than an absolute abundance. In the present study, we were primarily interested in the dispersal of *B. subtilis* instead of the absolute abundance. As expected, the abundance of *B. subtilis* from untreated samples was very low, only about 0.1–0.2% of those samples that received the BCA application. A recent study also showed strong positive relationships between visual disease symptoms and the relative abundance of the causal agents^26^. Thus, the relative quantification is unlikely to alter the conclusions regarding dispersal qualitatively. As this DNA-based technique can detect DNA from dead cells in the phyllosphere as well, the relative abundance of *B. subtilis* quantified represents the maximum relative abundance of viable *B. subtilis*. This should be kept in mind when discussing relative abundance in terms of bacterial survival/reproduction, particularly for the samples taken 8 days after the application.

There was a loss of 50% *B. subtilis* in the open field 8 days after leaves received the application; in contrast, no such loss was observed under the protected condition. This difference results most likely from the wash-off of bacteria by rainfall: several rainfall events occurred 24 h after application (but before day 8). Rainfall can dislodge and disperse conidia from substrates as well as aid in the dispersion of propagules. *Candida sake*^27^ and *Bacillus amyloliquefaciens* strain TrigoCor^28^ were easily rinsed off the plant surface within several hours. Rainfall as a dispersal agent for plant pathogens has been well studied^29^,^30^. Limited research has been done to study the effect of rainfall on the dispersal of microbial BCAs. Simulated rainfall significantly reduced *Bacillus thuringiensis* var. *Kurstakii*^31^, entomogenous fungi *Metarhizium anisopliae*^32^ and *Beauveria bassiana*^33^ from plant foliage. There is, however, no reason to believe that dispersal of microbial BCAs should be significantly different from the dispersal of bacterial and fungal plant pathogens. Similarly, lower abundance of *B. subtilis* on the ‘New’ leaves that emerged after the application, under the protected condition relative to the open condition, can also be explained by the lack of rain-dispersal under protection. Dispersal of BCAs between leaves under protection was achieved primarily from physical contact between leaves.

Present results suggest that BCA abundance as a proxy for the net outcome of survival and reproduction may not be the main reason for variable control results, particularly under the protected conditions. This is in contrast to previous results showing that BCAs declined to a lower level rapidly after several days^5^,^12^. It should be noted that leaf expansion may still result in a considerable reduction in population densities despite only a small reduction in abundance. Another study showed that *T. atroviride* can survive for 45 days on strawberry leaves under greenhouse conditions (25 ± 2 °C, RH = 60 ± 10%)^11^. Furthermore, BCAs (including *B. subtilis*) also have been reported to maintain high inoculum on fruit surface during long periods of postharvest storage^34^,^35^. More successful biocontrol of post-harvest diseases in storage may thus be explained by the fact that the host volume does not change following BCA application and hence there are no new tissues to protect.

Rapid losses of BCAs and limited dispersal to new tissues in open conditions suggests that repeated application (often at a short interval during fast host growth) is necessary to achieve commercially acceptable efficacy, negating an advantage of using microbial BCAs – they can reproduce and spread to new tissues and hence require fewer applications than synthetic fungicides. There are limited losses in BCA under protection but on the other hand there is limited dispersal of BCA to new leaves. This poses a dilemma in designing control strategies. We may argue that for tunnel crops BCAs are most likely to achieve better control during slow plant growth at a given application interval. However, during the fast growth period, application intervals may have to decrease in order to ensure most new leaves are protected by BCA. When considering controlling fruit diseases, particularly those originating from infection of flowers, the present results explain why there is lack of success in biocontrol. Blossoms of strawberry are well separated from each other and from leaves; we would expect virtually no dispersal of BCAs from leaves to flowers and among flowers, particularly under protection. Furthermore, new flowers continuously open over a long period of time, e.g. 4–5 months for day-neutral strawberry where botrytis control is most difficult, but infection of flowers by *Botrytis* occurs continuously (since it does not depend on a single critical event, like rainfall^36^,^37^,^38^). Thus, application of Serenade at an interval of 7–10 days (current commercial practice) will leave a large proportion of flowers unprotected. Bees could disperse BCAs from flower to flower, e.g. *Pseudomonas fluorescens* A506 and *Erwinia herbicola* C9–1S on apple blossoms^39^. However, bee activity is often weather-dependent and their effect on botrytis development in strawberry fruit was at best limited as well as variable (Saville & Wedgwood, per comm.).

There were significant differences in bacterial and fungal abundance between plants grown in open field and under protection. Similar differential abundance of phyllosphere bacteria between indoor and outdoor plants was observed on lettuce and *Arabidopsis thaliana*^40^,^41^. Proteobacteria made a large contribution to the difference in bacterial abundance between the two environments. Different classes of Proteobacteria have been shown to be enriched on either laboratory- or field-grown plants^40^,^42^. For instance, the phyllosphere bacterial diversity contained relatively higher proportions of Betaproteobacteria on laboratory-grown lettuce, whereas, Gammaproteobacteria was enriched on the field lettuce^41^. For fungal OTUs, as found in the phyllosphere of the grapevine^43^, Ascomycota and Basidiomycota were the most abundant fungal phyla. However, it remains to be determined whether and, if so, how such differences in phyllosphere microbial composition affect biocontrol outcomes. The effect of BCAs on overall microbial composition and individual microbial abundance can be ignored, agreeing with previous findings^43^,^44^,^45^. Even fungicide treatment affects only a few OTUs^26^,^43^. A previous study demonstrated that BCA treatments altered microbial community diversity and abundance but it used low-resolution molecular techniques^46^. It is not surprising that the effect of BCA on overall microbial composition and abundance of individual microbes in the present study is minimal because this *B. subtilis* strain was selected for controlling specific pathogens. Nevertheless, this also raises questions about competition for nutrients and spaces with other similar micro-organisms as an important biocontrol mechanism. Of course, the extent of influence of an introduced BCA on resident microflora depends on its mode of biocontrol mechanisms and specificity of its target pathogens.

In summary, we showed that the dispersal of *B. subtilis* was very limited, particularly under the protected condition. Greater losses of *B. subtilis* and its increased dispersal to new leaves in open field conditions probably resulted from rainfall. These results suggest that limited dispersal is the main reason for variable (and often low) biocontrol efficacy under field conditions. Further studies are necessary to quantify the absolute amount of microbes and their viability, e.g. using qPCR techniques, in order to better understand the ecological characteristics of an introduced BCA. This understanding is necessary to improve deployment and to predict biocontrol outcomes.

## Methods

### Experimental design and sample collection

During the 2015 growing season, an experiment was carried out in May and repeated once in June. In the first experiment, application of Serenade was on May 28^th^ and in the second it was on June 9^th^ 2015. There were four treatments: BCA treatment and untreated control for strawberry plants grown in open field and under protection.

Serenade ASO (a commercial formulation of *B. subtilis* strain QST713, 1.042 × 10^12^ CFU L^−1^) was diluted with sterile deionized water (SDW) to 1% (vol/vol) suspensions that contained bacterial cells at ca. 10^7^ CFU ml^−1^. Strawberry (cv. Vibrant) plants in open field and under protection (polytunnel) were used; these two crop plantings were next to each other. Before BCA application, two youngest but unrolled leaves were tagged on each plant; the two leaves on each plant were sprayed with ca. 10 ml Serenade suspensions via a hand held sprayer. Control plants were not sprayed. One of the two tagged leaves was cut-off from the plant with surface-sterilized scissors 4 hours after BCA application (4H); the other tagged leaf was collected 8 days after (8D). Finally, a third leaf was also sampled from the plant on day 8 (New) – this leaf emerged after the spray and hence had not been directly exposed to the BCA application. For each type of leaf sample (4H, 8D, New) of each treatment, three composite samples of leaves were obtained, each with five leaves – one leaf from each of five randomly selected plants, and bagged into a polypropylene box.

### DNA extraction

Microorganisms were harvested in aliquots of 5 leaves in a 400-ml screw-cap bottle based on a published protocol^47^ with some modifications. The bottles were filled up to 200 ml with 1:50 TE buffer (1 M Tris, 0.5 M Na EDTA and 1.2% Triton diluted in the sterile distilled water) and shaken at 270 rpm for 10 min. The leaf-washings suspension was filtered with sterile cheesecloth and centrifuged (4,000 × g, 4 °C for 20 min), the supernatant was discarded by pipetting, and a pellet of each sample was immediately frozen and stored at −20 °C before DNA extraction. Before DNA extraction, cells were re-suspended in 500 μl MoBio PowerSoil bead solution, and DNA was extracted from the resulting pellets using the MoBio PowerSoil DNA Kit (MoBio Laboratories, Carlsbad, CA, USA) following the manufacturer’s instructions. The extracts were purified with GeneClean Turbo Kit (MP Biomedicals) following the manufacturer’s protocol, and quantified using a NanoDrop ND-1000 spectrophotometer (NanoDrop Technologies, Wilmington, DE, USA).

### Next-generation sequencing

Bacterial 16S rRNA genes were amplified in a PCR using primers 341F and 805R^48^. The fungi-specific primers ITS1^49^ and EK28^50^ were used to amplify the ITS region of fungal rDNA. These primer sets were modified at the 5′ end with overhang adapters (TCG TCG GCA GCG TCA GAT GTG TAT AAG AGA CAG - forward adaptor and GTC TCG TGG GCT CGG AGA TGT GTA TAA GAG ACA - reverse adaptor). The reaction was carried out in triplicate for each sample in 25 μl volumes containing 10 × buffer basic (Molezym GmbH and Co. Bremen Germany), 2 mM MgCl_2_ (Qiagen, Hielden, Germany), 0.2 mM dNTP (Invitrogen, Life Technologies, USA), 0.25 U Mol Taq basic DNA polymerase (Molezym GmbH and Co. Bremen Germany), 0.2 mM of each forward and reverse primer (Integrated DNA Technologies) and ca. 5 ng template DNA. The following PCR conditions were used: initial denaturation at 94 °C for 5 min, followed by 25 cycles consisting of denaturation (94 °C for 30 s), annealing (55 °C for 45 s) and extension (72 °C for 1 min) and a final extension step at 72 °C for 7 min.

PCR products were visualised by agarose gel electrophoresis. DNA amplicons were combined for each sample, purified using Agencourt AMPure XP beads (Beckman Coulter, USA), and modified by attaching Illumina sequencing indices using the Nextera XT 96 Index Kit (Illumina Inc., San Diego, CA, USA) by PCR. Following the index PCR clean-up, the amplicons were quantified with Qubit dsDNA HS assay kit (Life Technologies, USA) on a Qubit 2.0 Fluorometer (Life Technologies, USA), and the size determined with High Sensitivity NGS Fragment Analysis Kit (Advanced Analytical, Ames, IA, USA) on a Fragment Analyzer™ (Advanced Analytical, Ames, IA, USA). Amplicons were then pooled in equimolar proportions in a single tube with a 4 nM final concentration. Sample denaturation was then performed by mixing 5 μl of the diluted library and 5 μl of 0.2 N fresh NaOH and incubated 5 min at room temperature. Chilled Illumina HT1 buffer (990 μl) was added to denatured DNA and mixed to make a 20 pM library. The diluted and denatured amplicon library was then combined with a denatured PhiX library at an equimolar concentration at a rate of 20% and sequenced using Miseq Reagent Kit v3 (600 cycles) on the Illumina Miseq platform (Illumina Inc., San Diego, CA, USA) with 300 bp paired-end sequencing.

### Sequence processing

Raw sequences were de-multiplexed by the Illumina MiSeq and then processed by the UPARSE algorithm^51^, version 8.0:

1. For bacterial sequences, the following criteria were used to merge the forward and reverse sequences: (a) individual read length ≥230 bp; (b) at least 10 bp overlap; (c) maximum number of expected base error (MEE) of the merged sequence ≤2; and (d) the length of merged sequences ≥420.
2. For fungal ITS sequences, the forward sequences were all trimmed to the length of 200 bp with the MEE set to 1.0; all reads with length <200 bp were excluded.
3. All merged or trimmed sequences were combined from all samples to generate unique sequences together with their frequencies.
4. Unique reads were clustered into OTUs at the level of 97% similarity and a representative sequence for each OTU was generated; unique reads with <8 counts across all 72 samples were excluded from this clustering analysis (but these sequences were not necessarily excluded from the final OTU table, depending on the extent differences of these sequences from other sequences – see step 5). Only unique sequences with >7 counts were included in forming OTUs because preliminary analysis showed that the greatest number of sequences were mapped to OTUs (i.e., step 5) when only the unique sequences with >7 counts were used to form OTUs. During the clustering process, chimeras were removed and a final chimera check is performed after clustering.
5. All initially processed sequences (including those sequences that were not used in the initial clustering analysis, i.e. with <8 copies) were assigned to an OTU at 97% similarity to generate an OTU counts table (counts of each OTU in each sample).
6. Each OTU was assigned with a taxonomy identity using the representative sequence against two international databases: SILVA 16S rRNA gene database^52^ and the UNITE fungal ITS database^53^ at 97% similarity. The algorithm identifies an OTU to the genus level only and generates a probability value for correct identification at each taxonomical rank. Taxonomic identity was determined at the following threshold P-values: 0.95 for kingdom and phylum, 0.90 for class and order, and 0.85 for family and genus. For those OTUs with P-values less than the threshold P-value at a specific rank, they were classified as ‘Unknown’ at the specific level and those below.

### Statistical analysis

Three types of statistical analyses were performed: (1) initial exploratory analysis [diversity indices], (2) comparing abundance of individual OTUs between treatments, and (3) detailed analysis of *B. subtilis* abundance. All non-bacterial (plant chloroplast) and fungal (confidence level <2 for the kingdom rank) OTUs were excluded for all statistical analysis.

Individual sample diversity (i.e., α diversity) indices were calculated: the number of distinct OTUs observed per sample (Sobs), and Shannon and Simpson indices (both related to the frequency of individual OTUs within a sample). A resampling (i.e. bootstrap) scheme was used to estimate α diversity indices for each sample. A bootstrap sample was obtained via randomly sampling a minimum number of sequences from the sequences in each sample (i.e., rarefying at the rarefaction point of 25,000 and 19,000 for 16S rDNA and ITS sequences, respectively). A total of 100 bootstraps were conducted. Next, we calculated weighted UniFrac distances among samples (i.e. β diversity), which incorporates information on the relative relatedness of community members by incorporating phylogenetic distances between observed organisms in the computation^54^. The α and β diversity indices were calculated using the respective metagenomeSeq^55^ and Phyloseq^56^ packages in R (version 3.2.0 × 64). The β diversity was calculated from transformed data in order to adjust for differences in sequencing depth between samples: the counts data were first ln-transformed and then individual OTU values were expressed as a percentage of the total for the sample. Hierarchical clustering analysis was then applied to the UniFrac distance matrix with the Ward’s minimum variance method. Finally, principal component analysis (PCA) based on the correction of the transformed counts data was carried out.

To assess differential OTU abundance between open and protected plantations, and between BCA treated and control plants, we used the DESeq2 statistical package in R^57^. DESeq2 uses the negative binomial distribution as an error distribution to compare abundance of each OTU between groups of samples in the framework of generalised linear modelling. Raw counts data (as recommended) were used in the DESeq2 analysis. In addition, DESeq2 also implemented an algorithm to automatically filter OTUs before differential abundance analysis based on several criteria, including variance in abundance across samples and overall abundance level. The number of OTUs filtered out by this algorithm depends on the sample collection used in analysis. For comparing abundance between open and protected conditions (Site), replicate experiments (Expt), sample (Leaf) types, BCA treatment (BCA), and the interaction between Leaf and BCA were included in addition to the Site factor. Differential abundance analysis between treated and control samples was conducted separately for ‘8D’ and ‘New’ leaf samples; in addition to the BCA factor, Expt and Site were included as factors. To correct discovery rate associated with multiple testing, DESeq2 uses the Benjamini-Hochberg (BH) adjustment^58^. Statistical significance was determined at the 5% level (BH adjusted).

Finally, abundance data for *B. subtilis* were extracted from the OTU table and subjected to more detailed analysis. The rlog (the regularised log transformation) algorithm as implemented in DESeq2 was used to transform the original counts data. Standard analysis of variance (ANOVA) was applied to the data to assess (1) BCA application efficacy (‘treated’ vs. ‘control’ for the ‘4H’ samples), (2) BCA survival/reproduction (‘treated’ vs. ‘control’ for the ‘8D’ samples), (3) BCA mortality (‘treated’: ‘4H’ vs. ‘8D’ samples), (4) BCA dispersal (‘treated’ vs. ‘control’ for the ‘New’ samples), and (5) Dispersal loss (‘treated’: ‘8D’ vs. ‘New’ samples). Comparison (4) assessed whether dispersal actually took place; whereas comparison (5) assessed what was the difference between the potential and actual dispersal. Expt was included as a block factor, and Site and the five comparisons were as factors. Although the five comparisons were not all orthogonal to each other, the conclusions did not change irrespective of the order of the five comparisons introduced into ANOVA. Residuals followed closely to normal distributions.

## Additional Information

**How to cite this article**: Wei, F. *et al.* Dispersal of *Bacillus subtilis* and its effect on strawberry phyllosphere microbiota under open field and protection conditions. *Sci. Rep.* **6**, 22611; doi: 10.1038/srep22611 (2016).

## Supplementary Material

Supplementary Information

## Figures and Tables

**Table 1:** Summary of *Bacillus subtilis* expressed as mean number of reads per sample, percentage of the total reads per sample, and normalised reads via the rlog algorithm as implemented in the DESeq2.

Treatment	Original reads			Percentage of the total			Normalised reads		
	4H	8D	New	4H	8D	New	4H	8D	New
Control/Open	2	1	5	0.002	0.001	0.004	3.687	3.614	3.399
Control/Protected	2	2	1	0.003	0.002	0.001	4.342	4.170	3.924
Serenade/Open	3,011	1,559	459	3.899	1.335	0.590	9.377	7.712	5.661
Serenade/Protected	1,017	1,151	70	1.062	1.352	0.087	10.627	10.148	6.487

Serenade (a commercially formulated *B. subtilis* product) was applied to strawberry leaves in open field or under protection; epiphytic microbes were profiled with Illumina MiSeq on leaves sampled (4 hours –4H, and 8 days –8D after application, and new leaves emerged after application - New).
