# Supplementary material for: Dispersal of Bacillus subtilis and its effect on strawberry phyllosphere microbiota under open field and protection conditions
Source: Sci Rep. 2016 Mar 3;6:22611. doi: 10.1038/srep22611 (PMC4776175; doi:10.1038/srep22611)
Supplement: Supplementary Information [file srep22611-s1.doc]

**Dispersal of *Bacillus subtilis* and its effect on strawberry phyllosphere microbiota under open field and protection conditions**

Feng Wei^1,2^, Xiaoping Hu^1^ & Xiangming Xu^2^

^1^State Key Laboratory of Crop Stress Biology for Arid Areas, College of Plant Protection, Northwest A&F University, Taicheng Road 3, Yangling 712100, China

^2^East Malling Research, East Malling, Kent, ME19 6BJ, UK

Correspondence and requests for materials should be addressed to X.H. ([xphu@nwsuaf.edu.cn](mailto:xphu@nwsuaf.edu.cn)) or X.X. (xiangming.xu@emr.ac.uk)

1. (b)

**Fig. S1.** Rarefaction curves of 72 samples for bacteria (a) and fungi (b) from strawberry leaves of cv. Vibrant with or without application of Serenade^TM^ (a commercial formulated product of a single *Bacillus subtilus* strain) in the open field or under protection. All curves terminated at the number of sequences obtained in the samples.
